# Promoting well-being through group drumming with mental health service users and their carers

**DOI:** 10.1080/17482631.2018.1484219

**Published:** 2018-07-10

**Authors:** Sara Ascenso, Rosie Perkins, Louise Atkins, Daisy Fancourt, Aaron Williamon

**Affiliations:** a Centre for Performance Science, Royal College of Music, London, UK; b Faculty of Medicine, Imperial College, London, UK

**Keywords:** Well-being, group drumming, music, mutual recovery, positive mental health

## Abstract

**Purpose**: Music has been linked with well-being across clinical and community settings. Yet, research has focused on assessment of single dimensions of well-being and on the typical receiver of support services. Acknowledging the burden that a caring role encompasses and integrating recent proposals for a multifaceted definition of well-being, we explore the extent to which group drumming interventions translate into multidimensional well-being change for both mental health service users and carers. **Method**: Thirty-nine participants engaged in one of a series of community drumming programmes were assessed via semi-structured interviews (n = 11) and focus groups (n = 28) at the end of each programme. Data were analysed using IPA. **Results and Conclusion**: Emotional, psychological and social dimensions of well-being emerged for both patients and carers, accounted for through six themes: (1) hedonia: positive affect and pleasant physical effects of drumming; (2) agency: initiative and sense of control; (3) accomplishment: non-specific and in relation to musical goals; (4) engagement, through focus and flow; (5) a redefinition of self, through self-awareness, construction of a positive identity, self-prospection and incorporation of a musical identity; and (6) social well-being, through connectedness and positive relationships. The potential of such interventions for clinical contexts is discussed.

## Introduction

The positive effects of music on well-being have been widely investigated (MacDonald, Kreutz, & Mitchell, 2012). Research has centred around two main threads: (1) the impact of *listening to* music and (2) the impact of *making* music. Studies focused on *listening to* music have evidenced a positive influence on stress reduction (Pelletier, 2004), emotional regulation and personal mood management (DeNora, 2000; Hays & Minichiello, 2005; Sloboda, 1999), pleasure and the arousal of positive emotions (Gabrielsson, 2011; Laukka, 2007; Salimpoor, Benovoy, Larcher, Dagher, & Zatorre, 2011), social bonding (Nilsson, 2008), and enhancement of cognitive functioning after neurological impairment (Särkämö, Tervaniemi, & Laitinen, 2008). Additionally, listening to music has been linked to an attribution of meaning in daily life for vulnerable populations such as older adults (Hays & Minichiello, 2005). Studies on *music making* have also highlighted its benefits for well-being. Group singing interventions have pointed to increases in general well-being (Clift et al., 2010), self-esteem, social bonding and meaning (Cohen et al., 2006; Davidson, 2011; Tarr, Launey, & Dunbar, 2014). With older adults, interventions focused on engaging with musical instruments have provided evidence towards anxiety reduction (Hars, Herrmann, Gold, Rizzoli, & Trombetti, 2014), a sense of accomplishment (Perkins & Williamon, 2014), purpose, control, autonomy, social well-being (Creech, Hallam, Varvarigou, McQueen, & Gaunt, 2013), and increased cognitive function (Seinfeld, Figueroa, Ortiz-Gil, & Sandrez-Vives, 2013).

In particular, the potential of group drumming to enhance well-being has been well documented. Winkelman (2003) highlighted drumming as an effective complementary tool for addiction treatments as it demands a reduction of alienation through connectedness with self and others. Camilleri (2002) pointed to drumming as a tool for creating a sense of community in underprivileged neighbourhoods, and Burnard and Dragovic (2014) demonstrated drumming’s potential to enhance well-being in educational contexts by facilitating a sense of empowerment, and through the embodiment inherent to music learning. Furthermore, studies with at-risk young people, alienated from the school system (Faulkner, Wood, Ivery, & Donovan, 2012; Wood, Ivery, Donovan & Lambin, 2013) highlighted the effects of drumming on social learning outcomes, including emotional control, improved relationships and increased self-esteem, when combined with cognitive behavioural therapy. Within mental health settings, drumming has proven effective as a tool for psychosocial rehabilitation of psychiatric inpatients (Tague, 2012), in burnout reduction for staff (Newman, Maggott, & Alexander, 2015), and in alleviation of depression and anxiety while enhancing well-being and social resilience among mental health service users (Fancourt et al., 2016).

Despite this evidence, diverse conceptualizations of well-being permeate the existing literature. A clear definition of well-being, which can be operationalized in research, is key in setting the context for further investigation in this field.

### Well-being and positive mental health

Research on well-being has reflected two complementary approaches, broadly referred to as the hedonic and eudaimonic traditions (Ryan & Deci, 2001; Waterman, 1993). The hedonic tradition typically places centrality on pleasurable experiences as the pathway to happiness. Well-being is concerned with the balance between positive affect and negative affect, along with perceived satisfaction with one’s life (Diener, 2000; Diener, Suh, Lucas, & Smith, 1999). The eudaimonic tradition advocates that virtue and the fulfilment of human potential and self-realization are at the core of well-being (Deci & Ryan, 2008; Waterman, 1993). One of the most influential models within the eudaimonic tradition was developed by Ryff (1989; Ryff & Singer, 2008), who proposed six dimensions of psychological well-being: self-acceptance, environmental mastery, positive relations with others, personal growth, autonomy, and purpose in life. These elements stand as empirically distinct from hedonic components, and their investigation has strengthened the case for equating well-being as more than just “feeling good” (Keyes, Shmotkin, & Ryff, 2002; Ryff, 1989). It has also been highlighted how well-being is more than merely a private phenomenon. Individuals are part of social structures and communities and experience social tasks and challenges. Social well-being, therefore, encompasses the experience and judgment of one’s own social functioning and stands as a distinct component from emotional and psychological well-being. Keyes (1998, 2002) proposed five dimensions of social well-being: social contribution, social integration, social actualization, social acceptance, and social coherence. The joint work of Keyes and Ryff led to a combined definition of well-being incorporating: (1) emotional well-being (positive emotions and life satisfaction), (2) psychological well-being (consisting of Ryff's (1989) six dimensions), and (3) social well-being (consisting of Keyes’s five dimensions). This conceptualization bridged the gap between hedonic and eudaimonic models (Keyes, 2002; Ryff & Keyes, 1995). More recently, another proposal by Seligman (2011) also brought to the debate the two traditions together. The PERMA model proposes that well-being is found through Positive emotion, Engagement, Positive relationships, Meaning, and Accomplishment.

These models of well-being have strengthened the conceptualization of mental *health* as different from the absence of mental *illness*, sustained by a combination of optimal levels in different areas of functioning (Keyes, 2007; Seligman, 2011). This broader view is shared with the World Health Organization’s (WHO) definition of health as a “state of complete physical, mental and social well-being and not merely the absence of disease and infirmity” (1946, p. 1). Assessing well-being, therefore, means assessing not only the absence of ill-being but, crucially, the presence of positive indicators. There is a long tradition in assuming that mental illness and well-being stand as opposites and that the treatment of mental health symptoms results in a mentally healthy population. This, alongside an emphasis on the categorical classification of mental disorders, had led to a tendency to reduce patients to the sum of their problems (Corrigan, 2004; Keyes, 2002). Focusing on clinical populations, however, there is now strong empirical evidence reinforcing well-being and psychopathology as two related but separate continua (Keyes, 2005). One continuum reflects the presence or absence of *symptomatology*, while the other reflects the presence or absence of *well-being*. As Keyes (2005) has demonstrated, the absence of psychopathology is neither necessary nor sufficient to ensure that an individual lives a productive, fruitful, and balanced life. Conversely, an individual can simultaneously experience symptoms of psychopathology and maintain positive functioning and psychological flourishing. This “two-continua model” has been validated in multiple populations (Keyes, 2006; Keyes, Eisenberg, Dhingra, Perry, & Dube, 2012; Keyes et al., 2008) and using different measurements and conceptualizations of mental health and illness (Compton, Smith, Cornish, & Qualls, 1996; Greenspoon & Saklofske, 2001; Headey, Kelley, & Wearing, 1993; Suldo & Shaffer, 2008; Westerhof & Keyes, 2010). An important implication is that an individual is only completely healthy when he or she experiences both low levels of psychopathological symptoms *and* a good state of well-being. Moreover, positive mental health may have effects on individual and social functioning that are independent from the effects of psychopathology (Howell, Kern, & Lyubomirsky, 2007; Pressman & Cohen, 2005; Veenhoven, 2008). Crucially, both reduction of symptoms and promotion of well-being are needed for complete mental health.

Conceptualizing well-being as the presence of positive elements rather than the absence of symptoms has led to a plethora of *positive interventions* in clinical contexts, prioritizing strengths promotion. Such interventions have been shown to be justifiable in their own right, as well as useful supplements to mainstream psychopathology treatment (Bolier et al., 2013; Duckworth, 2005). They bring a new approach by changing the nature of the intended “treatment” group, subscribing to the argument that everyone, regardless of mental illness symptoms, can enhance their psychological functioning (Seligman, 2011) and therefore benefit from such programmes. This idea has been taken forward by Crawford, Lewis, Brown, and Manning (2013), who proposed what they term the “mutual recovery” framework. Here, recovery is defined as the potential for gaining a more meaningful and resilient life despite the presence or absence of existing indicators of mental illness. In promotion programmes, this translates into collapsing the standard direction of “clinician treating patient” in favour of a model of co-construction of well-being, through a mutual encounter. Hitherto unexplored empirically, Crawford et al.’s (2013) notion of mutual recovery foregrounds interventions based in communities and facilitated by *creative* practices, which may provide spaces of trust and empathy. Indeed, joining the literature reviewed above centred on music, creative practice with different art forms has shown promising results in alleviating symptoms across a wide spectrum of conditions (Choi, Lee, & Lim, 2008; Ritter & Low, 1996) and has also proven effective in reducing professional burnout among physicians, clinicians, and mental health workers (Brooks, Bradt, Eyre, Hunt, & Dileo, 2010; Logid, 2011). However, research on creative practices within clinical settings has typically targeted alleviation of symptoms and reduction of ill-being rather than an in-depth assessment of psychological resources and positive functioning, failing to integrate the recent conceptualizations of well-being. Furthermore, findings for patients and clinicians typically remain independent of each other. If indeed recovery is mutually built, there is a case for an integrated evaluation of results, with all systems involved.

The purpose of our study emerges from the gaps expounded above, namely the need to: (1) refine the definition of well-being when assessing the impact of music-based promotion programmes, towards conceptualizing it as multidimensional positive mental health; (2) expand the investigation on the potential impact of participatory music-making on well-being, bringing together both mental health patients and carers; and (3) explore the specific characteristics of well-being that can be the result of a mutual creative encounter.

## Methodology

### Participants

Our study’s sample consisted of 39 participants as described in Appendix 1 (n = 11 men, n = 28 women), including adult mental health service user patients (n = 30), informal carers and formal carers such as psychologists and counsellors (n = 6), and participants who identified as both patients and carers (n = 3). Among the participants there were four dyads of patient-carer, who attended the sessions together. Recruitment was carried out through U.K. National Health Service (N.H.S.) practices, private practice referral, charities, community centres and carer online networks. For inclusion, participants needed to be over 18 years of age, currently accessing a mental health support service or having received that support in the past five years, or, alternatively, be acting as a formal or informal carer to a mental health patient.

The sample was drawn from a total of 61 mental health patients and carers who engaged in a wider study (64% of the total study population) (see Procedure below). Sampling was purposeful and targeted variation in sex and role (patient/carer). The study was granted ethical approval by the N.H.S. Research Ethics Service [13/LO/1811] and was conducted according to the ethical guidelines of the British Psychological Society (B.P.S.). Informed consent was obtained from all participants, and no payment was given in exchange for participation.

### Procedure

The choice of a music intervention was guided by Crawford et al.’s (2013) suggestion that creative practice may facilitate mental health recovery among patient and carer groups. Specifically, we designed and delivered a drumming intervention, as drumming is a practice that does not require knowledge of musical notation, making it particularly appropriate for a heterogeneous group with varying levels of prior musical engagement. Previous studies have shown the potential of group drumming for well-being promotion among vulnerable populations (Tague, 2012; Wood et al., 2013). Over the course of a year, four group drumming programmes were provided free of charge in West London, U.K., over either 6 or 10 weeks. Each weekly session lasted 90 minutes and was led by a professional drummer, assisted by 3 students from the Royal College of Music (R.C.M.). The room was set in a circle, with the facilitator and assistants positioned among the participants, and djembe drums were provided each week. The workshops were designed to be inclusive, based upon aural learning and consisting of call and response exercises and simple rhythmic patterns that built into larger pieces. At the end of each programme, an informal concert for friends and family took place at the R.C.M. Each group comprised between 15 and 20 members.

### Data collection

A qualitative approach was adopted in order to understand the subjective meanings, complexity, and nuances that a multidimensional construct such as well-being implies. Two interview formats were employed for data collection: one-to-one interview and focus groups. Semi-structured, one-to-one interviews were conducted with 11 participants within a week of completing each drumming programme. The interview schedule (Appendix 2) incorporated five main areas: (1) general evaluation of well-being; (2) evaluation of the group drumming programme; (3) drumming and feeling well; (4) drumming and functioning well; (5) drumming and recovery. Questions for components 3, 4 and 5 were informed by the areas of well-being suggested by Keyes (2002) to target multidimensional well-being assessment, but sufficiently broad and flexible to allow for participants’ idiosyncratic inputs and meanings to drive the interview. Interviews were conducted in a location and at a time convenient to the participant, lasting around 50 min. They were audio-recorded with permission and transcribed verbatim. All were face-to-face interviews, with the exception of one which was conducted over the telephone. It was possible to complement the interviews with 4 semi-structured focus groups involving the remaining participants (n = 28). Focus groups took place in the week following the completion of the programme and lasted between 32 and 67 min. The groups consisted of 6–10 participants facilitated by one member of the research team. The schedule covered the same five broad areas as the semi-structured interview (see Appendix 3). The discussions were audio-recorded with permission and transcribed verbatim.

### Analysis

Interview and focus group data were analysed through interpretive phenomenological analysis (IPA), which implies a double hermeneutic: making sense of the way participants make sense of their experiences, capturing their essence and meaning-making (Eatough & Smith, 2008; Smith, Flowers, & Larkin, 2009). Analysis was both theoretical and descriptive: informed by Keyes’ (2002) model of multidimensional well-being while also remaining driven by the content imparted by participants, retaining IPA’s commitment to idiography. It proceeded in five steps and was conducted using the qualitative analysis software NVivo 10. First, all transcripts were read multiple times for familiarity before, second, emergent meaning units were noted for each transcript on the left-side margins. These were also labelled electronically in NVivo. Third, the meaning units were clustered to form emergent sub-themes that denoted changes in well-being. Fourth, the sub-themes were integrated into a table of overarching themes and sub-themes for each individual participant and focus group before, fifth, after each transcript had been closely scrutinized, individual tables were integrated into one overall table capturing the study’s final overarching themes and sub-themes (Data from interviews and focus groups were analysed separately. Emergent themes were regularly revisited and discussed within and across transcripts. As the themes were highly convergent, the two sets were merged into one overall table). All stages of the analysis were conducted independently by two researchers and cross-checked at each stage to ensure agreement and a valid representation of the raw data. Final themes were also discussed with the broader team.

## Results

IPA revealed 6 overarching themes and 14 sub-themes as summarized in Table I. There was high convergence of themes across groups, with reports of change covering the same dimensions of well-being for both patients and carers, as well as for those in the 6- and 10-week programmes. In what follows, each overarching theme is explored in relation to its constituent sub-themes. Example quotes encapsulating the meaning of each sub-theme are presented from both the individual interviews and the focus groups, drawing upon both patients and carers.

**Table I.** The characteristics of wellbeing: overarching themes and sub-themes.

Overarching Themes	Sub-themes
1. Hedonia	1.1 Feeling good
	1.2 Physical effect
2. Agency	2.1 Initiative
	2.2 Control
3. Accomplishment	3.1 Overall accomplishment
	3.2 Musical accomplishment
4. Engagement	4.1 Concentration
	4.2 Flow
5. Redefined self	5.1 Self-awareness
	5.2 Positive identity
	5.3 Self-prospection
	5.4 Musical identity
6. Social well-being	6.1 Connectedness 6.2 Relationships

### Theme 1: hedonia

Feeling good (sub-theme 1.1.), or *hedonia*, was recurrently reported by all participants as a central characteristic of change resulting from the drumming programme. The sessions were associated with happiness, joy, fun, and a sense of being uplifted:

All that horribleness was drained out of you … I can’t even describe it […] it comes in beautiful flowing in you … I always left on a high. (Informal carer, interview)

It makes me feel euphoric, that’s the word, when I’m starting to play […] everyone felt the same uplifted feeling. (Formal carer, focus group)

It helps the stress, and you’ll come out feeling very happy, and it’s just a different happy […] I felt it inside and that’s why it was just really a wonderful and happy experience. (Patient, interview)

This heightened positive affect was also reported to outlast the time of the session. Anticipation was highlighted in this context: the programme promoted recurrent enthusiasm and joy to the participants’ days and the effect on positive emotion was manifest both right after the drumming and in the days preceding the following session:

When it ended I would still be on a high on that day. (Patient, interview)

The effect of the morning probably carried on for a day or two. (Formal carer, interview)

I looked forward to it during the week. […] On Sunday night I was already thinking of Wednesday […] it was something I always looked forward to by Monday evening, it was like kind of countdown for Wednesday morning. […] Definitely from Tuesday till Friday I am very happy, so I feel before the Wednesday class and a few days after, definitely happy. And my kids have noticed that. They would say, “Oh you’ve got your African drumming on Wednesday”, then you know, smile just lights up and my eyes apparently light up. (Patient, interview)

Finally, the experience of enhanced positive emotions was also reported as a shared experience by the patient-carer dyads. Hedonic well-being was experienced individually and in relation, both for informal care dyads as well as for professional support dyads:

I would be dancing in the street afterwards … me and mum with the rhythms stuck in our head and we even went to Marks & Spencer’s to get a sandwich and we were dancing at the till and made the man at the till laugh and so it was good … and it felt good that you’d made someone else smile yeah … just spread the really nice feeling […] excited and happy, and enthusiastic and glad to be having some quality time with my mum. (Patient, interview)

I could see the joy that she got out of it, so that’s quite an emotional thing for me […] I was very proud of her […] it lifted her, and I just thought to myself quietly, “I’m so glad that at some point we got involved in this because it´s kind of filtered out into my family.” That is really something I will cherish. (Informal carer/patient, interview)

It was really something [the client] was always looking forward to, it was like a highlight of the week many times, so I could see that and yeah, did it affect our work? Yes! […] our relationship did get better, different. (Formal carer, interview)

Accounts of positive emotions were frequently linked with references to the physical effect of the sessions, through repeated smiling and the specific impact of drumming in the body (sub-theme 1.2). The bodily engagement in drumming was linked to vitality and to “feeling good physically”:

My cheeks would hurt because I would feel the smiling. Yeah, I really did … I haven’t really smiled like that for a very long time. (Patient, interview)

To me it was about bringing myself more to the realm of music and my *body*! Because it made an impact on my balance and that´s in fact something that I am quite keen to continue, to keep the balance, because I can easily spend too much time in my head. (Formal carer, interview)

This sub-theme also emerged in relation to an experience of absorption of energy and a release of both energy and tension. Additionally, the physical demands of the sessions were reported to cause a sensation of tiredness which was typically positive:

It was being on the edge of [the leader]’s energy, it was like being on a wave, he generates such energy and somehow you can absorb that. (Informal carer, focus group)

It was a release of energy and anxiety. There were a couple of times when [the facilitator] said you can hit the drum hard if you like … and the drum became someone’s head. (Patient, interview)

Even if I felt equally tired, it was sort of a live tiredness rather than a numb, dead, detached tiredness. (Patient, focus group)

I felt it quite physical … I felt great coming out … so it works yeah! […] I got a lot out of it, I always felt better, sometimes tired, which is a good thing. Yeah … good tired! […] the long session we drummed the whole hour, didn’t we? I was beginning to perspire, that’s good!. (Patient, interview)

### Theme 2: agency

A second overarching theme accounted for increased agency. All participants reported enhanced proactiveness and a greater ability to act on their own will and make free choices. Here, there were recurrent references to new behaviours denoting initiative (sub-theme 2.1), occurring at three levels. First, the attendance of the sessions themselves was reported as a first step of intentionality and autonomy:

I felt good about making it to all of the sessions and being on time, because it has been a real issue for me in the past … so it was nice that I could see some improvement. […] It meant that I got up and did stuff. Because a lot of the time I won’t get up and do anything. (Patient, interview)

I think that was my goal really, that if I can achieve getting to a lesson feeling emotional, or when it was bad timing […] somehow I just knew that if I could achieve that goal then I can achieve other goals. (Formal carer/patient, interview)

Secondly, enhanced proactiveness was reported in relation to general openness and life engagement, including behaviours outside one’s comfort zone, such as signing up for new courses, starting a job, or connecting with others and being more available for social interaction:

When you do something you’ve never done before, and a lot comes out that you didn’t expect, you then realize that well, let’s try something else […] so there’s another little avenue, isn’t it, of possibilities. […] So I think I’ve been feeling actually able to think about doing things past the edge of where I’d normally think about doing things. (Informal carer/patient, interview)

It forced me to go: “What will I do?” So at the same time as doing the drumming, I started college! In a construction course. I’m volunteering with an animal hospital. So it’s brought me out of my shell […] enjoying the opportunities that come your way and saying yes to things. (Patient, interview)

I found it was very therapeutic to me to get out of my zone that I normally keep in, away from people. (Patient, interview)

Before I felt a bit more withdrawn and right now I will kind of say, “Okay, I’ll meet up … yes I will meet up”, and people have noticed a little bit that change in the past 6 weeks. (Patient, interview)

Thirdly, initiative was also recurrently mentioned in relation to musical endeavours (e.g., listening to music in the car and buying a drum):

I still do it and I think the nice thing was a couple of the girls I already knew […] one of them has a couple of djembes and my mum is going to get me one for my birthday and we are probably going to meet up in the park and do some playing … because we all really want to keep on playing. (Patient, interview)

Across the three threads within initiative, the theme emerged related to both engagement in new activities and projects, and with the recovery of old habits that had been lost after the onset of the mental health condition:

So, today, I was trying to remember the rhythm, and I was just doing that on the steering wheel. So, again I think that’s brought back my old personality that I used to have […] I used to love music, I just, I don’t know, I didn’t get around to it … And now, I have the radio and I start to feel the rhythm a bit more and I’m getting my old music out, and I started doing that. (Patient, interview)

Within the overarching theme of agency, another recurrent thread accounted for an increased sense of control (sub-theme 2.2). Participants highlighted the importance of regular commitment to giving structure and a perception of being in charge of life. This was captured through two threads of meaning. First, the routine of the sessions was understood as a means to higher regularity and sense of structure in daily life:

I think the routine of every week going somewhere, much more in myself, so much more let’s say, realizing that routine […] working as a team or being in groups really does hold the days together and the weeks together and the months together. […] I think that’s important for mental health and well-being because it is a bit of a glue, isn’t it? (Patient, focus group)

I feel quite in control of the way my life is running, and I would say that’s different from how I felt when I saw you ten weeks ago. It’s a definite commitment and it was a very good structure to my week. (Informal carer, interview)

That’s been great because it´s going somewhere, punctuating my week. […] And nothing got in the way in those ten weeks that was more important than coming to drumming for me. (Patient, interview)

Second, participants reported a higher sense of control over life through generally feeling confident and empowered:

[It gave me] motivation and I believe a little bit more in myself that I can! The same way I could achieve coming to this class and learning […] I just felt I was quite numb and in a sleepy mode … I didn’t really feel I could do it […] It’s interesting that I do *feel* a bit more ready. I’m trying to go more to the gym again. I just feel more positive or I’m making better decisions, which before I felt was more drowned into the negativity and feeling sorry for myself and not being able to cope, and now I just say “Right, I’m not in the mood for the gym, I’m in pain but I’m going to go because I know I’m going to come out feeling better.” (Formal carer/patient, interview)

I’ve put the hammer away in the handbag. It’s gone, do you know what I mean? I’m inviting life in […] I am living 50% chaos and 50% organizational skills at the moment. I’ve gained the time-management skills. (Patient, interview)

### Theme 3: accomplishment

A third overarching theme was the experience of accomplishment. This emerged on two levels. First, participation in the drumming was found to bring a sense of achievement, gratification, and triumph (sub-theme 3.1). This was highlighted in association with broad aims such as making it to the session and not giving up (in relation with Theme 2):

“Can I cope with this? Am I pushing myself too much?” But the very thought that I just did it that day … Just the fact that I dragged myself there! It means it was something I wanted to do and knowing the reward was going to be an energy coming back which then I could put into the group. I feel accomplished and there’s nothing like it, is it? I’m not horizontal in a bed and feeling like absolutely lousy about myself. It’s just a self-perpetuating situation. Lethargy brings lethargy. Energy will bring energy. I definitely feel accomplished. (Patient, interview)

There is a lot because […] it gives me a new view, I survived it so that goes in, it’s like knowing you do something and you come out the other end, you can go “Oh!” (Informal carer/patient, focus group)

It’s the sort of seed of thinking “OK, well, I have had the experience of being completely lost and then getting it! (Formal carer, focus group)

The sense of overall achievement also appeared strongly linked with a sense of group identity: achieving through journeying and succeeding together, as a group:

To show … to give something … and yeah to feel good about something [smiles] a little achievement … together we could create something else, it was a sense of achievement! (Formal carer, interview)

I felt a sense of achievement because I think as a group we did really well and it wasn’t easy stuff so I felt good about that […] even though the people there are much more physically, I don’t know if disabled is the right word, but they would not be hampered by their problems … I didn’t feel embarrassed about it; it was like more unifying […] well screw what everyone else thinks because I’m in a room full of really cool people and they are really nice. (Patient, interview)

The accepting and forgiving learning framework of the sessions and the inclusiveness of drumming also emerged in relation to the sense of achievement. Participants highlighted a redefinition of what counts as success and the freedom of the learning approach in this context as a means to fulfilment:

This was life-changing! Because I come from a background of failure, and shame and disappointment. I find learning in the formal way, how people learn, absolutely impossible. So when you take that framework, you have people that are not being graded, not performing to anybody […] you get this liberty. I found it really powerful, and the first session in a word I would say surprising [about] not only how much I could do but how good everyone else was … […] we were creative. It was a good standard, it was a good sound! (Patient, interview)

I like the way it didn’t matter if you were getting it, or you don’t have to do all the beats […] you’re not feeling left out and feeling like you’re a failure. (Patient, focus group)

That’s the nature of my personality. If I’m going to do something, I want to get it right. A part of what I’ve learned from this is that there are different ways to getting something right. (Patient, interview)

A second sub-theme within the overarching theme of achievement captured experiences of contentment reported as directly linked with musical processes and goals (sub-theme 3.2.). The final performance was especially highlighted in this context:

I felt fulfilled on kind of a … I’m trying to think of the right word … in a way that I’m interested in music and I’m interested in rhythm and that and I’m interested in making my own music and stuff, so I felt like I was stretching myself in that direction. (Patient, interview)

I was quite pleased with myself! Like a party in your head, because you got it right you know? Yes, when you got the rhythm right! (Patient, interview)

You have those crystallized moments […] to have that moment, *feel* it and know you might be drumming for another three years before you have another one. It has that kind of feel whenever you are learning something … you hit it and you kind of go “Yes!!” So that’s quite a little gem for my little gem box. (Informal carer/patient, interview)

The specificities of drumming that optimize the sense of accomplishment were also highlighted, especially the easiness in producing sound, regardless of one’s skills:

I think it’s very hard to go into a singing group, when it feels like “Oh gosh I need to sing in tune, I need to …” whereas bang, and you just bang. It’s fine! (Formal carer/patient, focus group)

They go “Oh great … I’m taking a sound out of that” … You’re right to choose percussion because you can’t get the sound out of a … I mean a piano takes you 30 or 40 minutes to get a sound of … you know […] you get a sound out straight away […] it was just wonderful. (Patient, interview)

### Theme 4: engagement

The drumming sessions were reported as a means for enhanced focus and concentration (sub-theme 4.1). Acoustic features were often highlighted in this context (regular beat and vibration). The engagement experience was reported as holistic, involving mind and body, and as a means to escape intrusive thoughts:

It was quite therapeutic, because you have to really concentrate so, you can’t think about your problems at the same time. It was more a case of being absorbed for 5 minutes and then having some intrusive thoughts, and then being absorbed again. There were a couple of times where that didn’t happen, and I was completely absorbed in the drumming for most of the session, with only one or two intrusions. That did feel really good! Possibly one of the best experiences that I’ve had over the last 18 months. (Formal carer/patient, focus group)

I think you engage … you engage your soul … your body and your soul when you are doing that. And the energy that the sound creates, and the fact that you are also creating the sound too … the different experiences. (Formal carer, interview)

Linked with concentration there were accounts of moments of extreme involvement, denoting a sense of energized focus, suggesting experiences of *flow* (as conceptualized by Csikszentmihalyi (1990)(sub-theme 4.2). Participants highlighted the sessions as strongly immersive, frequently leading to a sense of getting taken away. The state of flow was associated with drumming in unity with a group for continued time and with fast tempos:

We all sort of lifted off the ground a little bit […] being in flow and completely taken up by something was really powerful. (Patient, focus group)

It was quite easy to get into trance because once you’ve done the drumming for a certain amount of time, collectively, you just go into that place where you are in the moment, you are not worried about the flyover or the noise or the workmen climbing up and down outside or whatever is going on outside … you are just there in the moment. (Informal carer, focus group)

When we were in the group altogether and drumming really fast … I think it’s a kind of alternative reality in a way, a different stream of consciousness. I felt it every time, when we were all in the group drumming, it was really … a different kind of state of consciousness in a way. If you are taken away […] yes, you have another reality, well for me anyway. (Patient, focus group)

Within this sub-theme, an altered experience of the sense of time was also highlighted:

I had no concept of time passing. (Patient, interview)

When you’re drumming, time is marked by the beat. It’s not marked by other things […] it depends on what we’re doing … but it might be marked by clocks or whatever else, or a schedule that you’ve got to go somewhere, an appointment, whatever else … but when you are in that room, drumming time seems to be marked by the beat … so it speeds up, slows down so maybe you naturally just lose yourself in that particular beat, rather than the clock. (Patient, focus group)

### Theme 5: redefined self

The fifth overarching theme accounted for changes in the way participants defined themselves. Through engaging in the sessions and, in particular, mediated by musical achievement (sub-theme 3.2) and the sense of control (sub-theme 2.2) came a widening of the perception of self and enhanced self-awareness (sub-theme 5.1.). Participants reported being more attentive to their own characteristics, realizing what they were capable of doing. Finally, self-awareness was mediated by the group and the awareness of others:

You can’t change who you are but I’m *aware*, and I’ve added on a little bit of equipment to help me understand myself a bit more. (Patient, interview)

It can help your personality … to find yourself! I think I learned again something about myself … that I can do it! (Patient, interview)

It was always about knowing what I was capable of doing. You don’t know unless you put yourself in these different situations. (Informal carer, focus group)

Closely linked with self-awareness, and with a sense of control (sub-theme 2.2), all accounts revealed a definition of self that was centred on strengths and the building of a positive identity (sub-theme 5.2):

I am quite happy with myself. I’m quite socially confident, I’m quite clear thinking and also, I sort of fit in well with quite a lot of different types of people. I enjoy my friends […] I enjoy a lot of activities, cultural, museum, galleries, theatre, cinema … *beauty*, I enjoy the *beauty*. I look for the beauty. (Patient, interview)

I am a person who wants to uplift myself and others. I want to inspire myself and others, and to keep learning, not go: “That’s it. I’ve learned everything now.” I think my music taught me that. You can never just sit on your laurels and go, “That’s it, I’m the most perfect person.” […] There’s always someone that comes along that will surprise you, and that’s the wonderful thing about this life. With all its tragedy, there is a fantastic colour out there, that I want to keep. […] I do like engaging with people. It’s nice to see that trait of mine hasn’t stopped because it’s going to serve me. It’s starting to already serve me well. (Patient, interview)

Symptomatology was absent from all accounts, and descriptions of self were geared towards perceived competence domains, new skills, proactivity and new relationships. The positive redefinition of self was also evident through a normalizing approach towards mental health conditions and references to patients as an outside group (with the use of “them” instead of “us”):

Not quite sure I’d call it an illness because I think everybody—I think it’s very human. I think everybody has that in them. (Patient, interview)

I know there were a couple of various … sick people who attended. They would communicate with us as well quite … yeah. So I think it’s a sense of group achievement of some sort. (Patient, interview)

In the end when [the facilitator] said we can play what we’ve learned, I was thinking “Gosh, have I … what have I … I can’t … I haven’t got each thing … but I mean, maybe other people haven’t either!” (Patient, interview)

The redefinition of self was also evident through accounts of self-prospection (sub-theme 5.3). Linked with the experience of greater initiative (sub-theme 2.1), participants highlighted how the different views of themselves were impacting the way they saw their future and how enhanced awareness triggered direction in life and contentment:

I learned something about myself and it tapped a happy part of me to look forward to. Looking forward to doing something for my future because that was my goal for, I think, a good year. I didn’t really feel I could do it, but now I feel that I am ready and I feel that I want to be more proactive to start looking for work, hopefully in counselling or anything really. I feel a bit more confident because I was quite fearful maybe to go for an interview … that people would see that I am, you know, depressed or going up and down, and right now yeah, I still feel a lot positive so I want to hang on to that really. (Formal carer/patient, interview)

Everything matters, we all have an existence that I think *is* a purpose! So, no matter what, keep going! […] I want to feel good because when you feel good, it doesn’t matter what it is we’re talking about, you can contribute. You can put yourself out there, there’s an energy. There’s a different energy and energy comes to you as well but just to get on with your venture of life. It doesn’t have any particular shape—my little happy house—not really! It’s about just being in the moment, living life and feeling comfortable. Having a comfortable feeling inside, not the uncomfortable anxiety feelings I’ve had before. […] I’m realizing what I can contribute and already that you just got to live it! You just got to get on with it, and I’m loving it again! My life has a little more direction. (Patient, interview)

A final group of specific themes linked with self, captured the construction of a musical identity (sub-theme 5.4): defining oneself as musical. This was true for both participants with and without a musical background:

I came back elated and of course I phoned all my family in South Africa, “Oh my God, you´re not gonna believe this! I can play!!!” You know, with the kids on Skype now I say “I’m into the music now, you know … I’m cool!” (Informal carer, interview)

We’re *musicians* now!! Oh, we’re drumming! We’re going to start playing the piano next. (Informal carer/patient, focus group)

The formation of a musical identity appeared closely linked with initiative (sub-theme 2.1), with participants reporting behaviours such as purchasing drums, listening to YouTube drumming videos, and offering the recordings of the drumming sessions to friends and family as Christmas presents.

### Theme 6: social well-being

Linked with agency (overarching theme 2) and positive identity (sub-theme 5.2) emerged frequent accounts of enhanced social well-being. A sub-theme within this context was the experience of group connectedness in the sessions (sub-theme 6.1). All participants reported experiencing a sense of belonging and a meaningful relational link with the group, even when verbal interactions were scarce:

The class felt supportive, even though I didn’t know the people, there was that connection with the music and the energy, and that’s something that I’m already having, today, withdrawal symptoms of … […] I didn’t meet everybody but I still felt a kind of connection and I felt it was through the music and through the work that we were doing in the session, really. (Patient, interview)

It was nice not to know who was who. So we didn’t have to put a label, “This one is a therapist, this one is a patient” … and obviously some people you could see they were in different needs … but it was nice actually not to know and to see the people were connected … because there *were* connections made there! […] When we managed to play together and to feel there was a … kind of a … the sounds and energy, very much like … like a *circle* … a circle! This thing works on another level. This is non-verbal and using the sounds and the music and again also I feel the group also carries another part of healing … it’s a healing I can see that. […] Yes we didn’t speak, we didn’t find out what they were up to or what they do or don’t do … even though sometimes we knew their names, sometimes we didn’t, so I felt that the music or what we were doing gave another way of relating. (Formal carer, interview)

The experiences of the programme were also reported to positively influence relationships (sub-theme 6.2). In the case of dyads (patient-carer), this influence started to be noticed within the dyad relationship. For some, this change was mediated by the increase in shared hedonia (as described in Theme 1). Other participants equated the programme as a space of shared interest or generally as a trigger for closeness:

It’s brought me and [daughter] together in a different way […] I’ve gained a huge—just a by-product—a huge moving forward with my daughter. (Informal carer/patient, interview)

It’s nice actually … to have something we both like together! Because I’m not about … the shops. (Patient, interview)

There were also frequent accounts of higher relatedness and increased comfort in social interactions outside the sessions. Here, the changes in self (Theme 5) and the inclusiveness and “normalizing” atmosphere of the group seem to have led to a greater sense of social acceptance and confidence:

I think it makes me feel more confident in dealing with other people because I’ve got more of a sense that everyone is lovely in their own way, and I’ve got my own sense that I’ve got my own stuff in the sense that “it doesn’t really matter if you don’t like me because I know who I am” kind of thing … I can say I’m feeling a bit more likeable just from getting on with other people … I feel a bit more encouraged that people are good natured. (Patient, focus group)

Overall, the impact of drumming on social well-being was experienced by all, both within the context of the sessions and in the participants’ wider social sphere.

In summary, group drumming offers a means for enhancing well-being for both mental health patients and carers tackling the three core elements of positive mental health (Keyes, 2002): (1) emotional well-being, (2) psychological well-being and (3) social well-being.

## Discussion

This study has shed light onto the characteristics of well-being elicited through group drumming, for both mental health patients and carers. Six emergent overarching themes accounted for the main areas of change and were shared by both groups. The integrated proposal of multidimensional well-being provided by Keyes (2002) helps further interpret our findings. First, participants reported greater emotional well-being, through the experience of hedonia: positive emotions and positive sensations from the physical effects of drumming on the body. Several characteristics of psychological well-being were also highlighted. First, agency, through initiative and an increase in the sense of control in life, closely linked with the constructs of environmental mastery and autonomy (self-determination and self-regulation) proposed by Ryff (1989) and part of Keyes’ (2002) integrated model. Second, participants experienced a greater sense of accomplishment, both non-specific and in relation to musical goals, through the high intrinsic reward fostered by engagement in the sessions (implying focus and experiences of flow). The programme functioned as a seed towards a sense of continual development in achieving one’s full potential (what Ryff [1989)] named personal growth). Theme 5 accounted for a redefinition of self, through enhanced awareness, the construction of a positive identity, self-prospection, and the incorporation of a musical identity. Participants consistently expressed positive attitudes towards the self and towards their history. This was particularly reinforced with the normalizing language about mental illness. Self-acceptance, another element highlighted by Ryff (1989) and Keyes (2002), was therefore strongly represented in our data across the four sub-themes that build the theme of redefined self. Finally, drumming was a means for social well-being, through connectedness and the experience of positive relationships. This was evident both within the group, which was perceived by all as a relationally safe space, and, crucially, extended to social contexts outside the session. Within this area of well-being, three of the elements proposed by Keyes (2002) seem to have prominence in participants’ accounts: social acceptance (holding a positive view of the social world), social integration (seeing oneself as belonging to a social unit), and social contribution (seeing oneself as providing meaningful input socially). Lastly, the accounts on agency, accomplishment, and optimized relational processes seem to be denoting a contribution towards a greater sense of direction in life for these participants, tackling the element of purpose and meaning.

These results reinforce previous accounts of hedonic well-being through music-making (DeNora, 2000) and of group music learning as a means for engagement and accomplishment (Perkins & Williamon, 2014), extending the confirmation of these effects to a clinical population. In addition, our results highlight the potential of group drumming as a valid tool for promoting social well-being, suggesting that changes in well-being through music can go beyond the private individual level, and are sufficiently empowering to build strong connectedness and promote openness, agency, and social engagement, in line with suggestions by Wood et al. (2013). This seems particularly relevant in light of the centrality given to relationships and social participation in key models of well-being (Keyes, 2002, 2007).

The multidimensionality of change emerging from the data was convergent for all groups. In addition to strengthening the value of drumming for promotion of multiple components of well-being, these results underline that the scope for optimizing psychological functioning through music interventions is not dependent on diagnostic status or role. Both patients and carers experienced increased well-being as a result of drumming together, manifested through the same elements and evidencing a self-reported “recovery” of previous levels of functioning. Here, the findings lend empirical support to Crawford et al.’s (2013) conceptual framework which proposes that “recovery”—meaning more than the absence of symptoms and encapsulating the development of higher well-being—can be experienced by different participant groups when they come together in a mutual, creative space. Group drumming appears as a highly useful tool to facilitate this process.

Specific characteristics of group drumming may be responsible for potentiating the multidimensional change observed throughout this programme, as described fully in Perkins, Ascenso, Atkins, Fancourt, and Williamon (2016). Indeed, drumming enables an equalitarian setting, where all participants have the same function. The experience of redefining roles from a top-down “provider-receiver” framework to a “fellow musician” experience seems particularly empowering and may lay the foundation for the mutuality of recovery. This was highlighted by both patients and carers through their accounts of accomplishment linked with the group and through musical and social connectedness. Additionally, a drumming group does not require *a priori* musical skills; participants can contribute with different levels of sophistication according to their capability, allowing for an appropriate level of challenge through various degrees of proficiency. This brings a highly inclusive environment for learning and fosters motivation. Drumming also does not require verbal engagement, allowing for establishing bonds independently of more traditional social skills. Finally, exploring group drumming in relation to other settings of mutual support within mental health settings can bring further insight. Contrary to the experience of clinical mutual-help groups (Pistrang, Barker, & Humphreys, 2008) where participants typically have a similar diagnosis and specific goals regarding a common condition, an all-inclusive drumming workshop allows for a shift from the condition itself, to the music-making as the main agenda. Instead of focusing the dialogue on a shared problem, attention is directed towards engaging in creatively co-constructing a common output. The element that stands as the base for the bond is therefore highly positive.

### Limitations and further research

Despite providing evidence of the impact of group drumming interventions on a wide span of well-being characteristics for both mental health patients and carers, this study did not allow for an assessment of sustainability of the effect over time. Follow-up research is needed to track the intervention’s long-term impact. Furthermore, the design of the present study did not include an assessment of pathology. This would represent a highly valuable addition to the literature, allowing for a complete assessment of the potential of this intervention through the lens of the dual continuum model of mental health proposed by Keyes (2002).

Notwithstanding the above, this article provides evidence that group drumming programmes can lead to multidimensional enhancement in well-being among both mental health patients and carers. Indeed, the wide array of self-reported changes resulting from drumming tackled issues such as increased engagement, agency, and a redefinition of self which remain key goals in the context of therapy across varied mental health conditions (Adam & Sutker, 2001). This suggests that such initiatives may have potential as a valid complement to established clinical practices, also addressing concerns as to the mental health of caregivers (Morse, Salyers, Rollins, Monroe-DelaVita, & Pfahler, 2012; Shah, Wadoo, & Latoo, 2010). Participants’ reports on generalization of change to contexts outside the programme reinforce this potential. Given the process of resistance that is known to undermine therapeutic timelines in mental health support (Cormier & Nurius, 2003), another aspect that deserves attention is the time frame for self-reported change in the context of this project. A short-term drumming intervention was enough for a perception of recovery in all well-being components explored, suggesting that the potential cost-effectiveness of such initiatives also deserves close attention in future research.

## Supplementary Material

Supplemental Material

## Appendix 1. Participant characteristics

**Table:**

Participant	Status	Gender	Data	Programme
1	Patient	M	Interviews	6 weeks
2	Carer (informal)	F		
3	Carer (formal)/Patient	F		
4	Carer (formal)	F		
5	Patient	F		
6	Patient	F	Focus group	
7	Patient	F		
8	Patient	F		
9	Patient	F		
10	Carer (formal)	F		
11	Patient	F		
12	Carer (formal)	F		
13	Carer (formal)	M		
14	Patient	M		
15	Patient	M		
16	Carer (informal)/Patient	F	Interviews	10 weeks
17	Patient	F		
18	Patient	M		
19	Patient	M		
20	Patient	F		
21	Carer (informal)	F		
22	Patient	F	Focus group	
23	Patient	M		
24	Patient	F		
25	Patient	M		
26	Patient	F		
27	Patient	M		
28	Patient	M		
29	Patient	F		
30	Patient	F		
31	Patient	F		
32	Carer (informal)/Patient	F		
33	Patient	F		
34	Patient	F		
35	Patient	F		
36	Patient	M		
37	Patient	F		
38	Patient	F		
39	Patient	F		

## Appendix 2. Semi-structured interview schedule

**Table:**

Domain			Questions
Global wellbeing-evaluation			How would you grade your general wellbeing?
Evaluation of programme			How did you feel when you started the programme? How do you feel now?
			Tell me about your experiences in the sessions? What were the greatest moments? What, if any, were the challenges of this programme? Suppose that you were in charge and could make changes that would make the program better. What would you do?
Feeling well			During the past few weeks, how often did you feel happy?
			Tell me about most enjoyable tasks during the sessions
Functioning well		Personal relationships	Let’s talk a little about relationships. In the last few weeks, how satisfied are you with your personal relationships?
		Social adjustment	How was the experience of working in a group? Have you noticed any difference in your interactions with others since the programme started?
		Self-concept	Imagine you had to describe yourself to someone that just met you, what would you say?
		Meaning	During the programme, how often did you feel that you had experiences that challenged you to grow positively? Have you noticed any difference in the way you think about life recently?
		Accomplishment	What do you feel you have gained from these sessions? In the past month, how often did you feel good at managing the responsibilities of daily life?
Recovery/Mutual recovery			[If applicable]: How do you think this has helped you deal with (condition)? What do you think it is about the sessions that helps in that? How was the experience of having people from so many different backgrounds here?
Overall well-being			Taking all things together, how happy would you say you are these days?

## Appendix 3. Focus group protocol

**Table:**

Introduction	Welcoming participants; introductions Explaining purpose and context of the focus group (no right or wrong answers, keeping confidentiality within the group) Explaining about the research project and ethics, that information is confidential and no names will be used
Main body	General well-being evaluation: How would you rate your wellbeing these days? Evaluation of the programme: What is your general evaluation of this programme? How did this programme affect the way you feel day-to-day? How did you experience doing music as part of this particular group? [If applicable] Recovery: How does the making of music help us in this change ? Transition: Of all the things we discussed, what do you think is the most important aspect to take from this experience?
Close	Thanks; contact information for further follow up; explain how data will be used
