# Supplementary material for: Promoting well-being through group drumming with mental health service users and their carers
Source: Int J Qual Stud Health Well-being. 2018 Jul 10;13(1):1484219. doi: 10.1080/17482631.2018.1484219 (PMC6041820; doi:10.1080/17482631.2018.1484219)
Supplement: Supplemental Material [file ZQHW_A_1484219_SM0056.docx]

Table 1. The characteristics of wellbeing: Overarching themes and sub-themes

| Overarching Themes | Sub-themes |
| --- | --- |
| 1. Hedonia | 1.1 Feeling good |
|  | - 1. Physical effect |
| 2. Agency | 2.1 Initiative |
|  | - 1. Control |
| 3. Accomplishment | 3.1 Overall accomplishment |
|  | 3.2 Musical accomplishment |
| 4. Engagement | - 1. Concentration |
|  | 4.2 Flow |
| 5. Redefined self | 5.1 Self-awareness |
|  | 5.2 Positive identity |
|  | 5.3 Self-prospection |
|  | 5.4 Musical identity |
| 6. Social wellbeing | 6.1 Connectedness  6.2 Relationships |

*Appendix 1. Participant Characteristics.*

| Participant | Status | Gender | Data | Programme |
| --- | --- | --- | --- | --- |
| 1 | Patient | M | Interviews | 6-weeks |
| 2 | Carer (informal) | F |  |  |
| 3 | Carer (formal)/Patient | F |  |  |
| 4 | Carer (formal) | F |  |  |
| 5 | Patient | F |  |  |
| 6 | Patient | F | Focus group |  |
| 7 | Patient | F |  |  |
| 8 | Patient | F |  |  |
| 9 | Patient | F |  |  |
| 10 | Carer (formal) | F |  |  |
| 11 | Patient | F |  |  |
| 12 | Carer (formal) | F |  |  |
| 13 | Carer (formal) | M |  |  |
| 14 | Patient | M |  |  |
| 15 | Patient | M |  |  |
| 16 | Carer(informal)/Patient | F | Interviews | 10-weeks |
| 17 | Patient | F |  |  |
| 18 | Patient | M |  |  |
| 19 | Patient | M |  |  |
| 20 | Patient | F |  |  |
| 21 | Carer (informal) | F |  |  |
| 22 | Patient | F | Focus group |  |
| 23 | Patient | M |  |  |
| 24 | Patient | F |  |  |
| 25 | Patient | M |  |  |
| 26 | Patient | F |  |  |
| 27 | Patient | M |  |  |
| 28 | Patient | M |  |  |
| 29 | Patient | F |  |  |
| 30 | Patient | F |  |  |
| 31 | Patient | F |  |  |
| 32 | Carer (informal)/Patient | F |  |  |
| 33 | Patient | F |  |  |
| 34 | Patient | F |  |  |
| 35 | Patient | F |  |  |
| 36 | Patient | M |  |  |
| 37 | Patient | F |  |  |
| 38 | Patient | F |  |  |
| 39 | Patient | F |  |  |

*Appendix 2. Semi-structured interview schedule*

| Domain | | | Questions |
| --- | --- | --- | --- |
| Global wellbeing-evaluation | | | How would you grade your general wellbeing? |
| Evaluation of programme | | | How did you feel when you started the programme? How do you feel now? |
|  |  |  | Tell me about your experiences in the sessions?  What were the greatest moments?  What, if any, were the challenges of this programme?  Suppose that you were in charge and could make changes that would make the program better. What would you do? |
| Feeling well | |  | During the past few weeks, how often did you feel happy? |
|  |  |  | Tell me about most enjoyable tasks during the sessions |
| Functioning well | Socially | Personal Relationships | Let´s talk a little about relationships. In the last few weeks, how satisfied are you with your personal relationships? |
|  |  | Social Adjustment | How was the experience of working in a group?  Have you noticed any difference in your interactions with others since the programme started? |
|  | Personally | Self-concept | Imagine you had to describe yourself to someone that just met you, what would you say? |
|  |  | Meaning | During the programme, how often did you feel that you had experiences that challenged you to grow positively?  Have you noticed any difference in the way you think about life recently? |
|  |  | Accomplishment | What do you feel you have gained from these sessions?  In the past month, how often did you feel good at managing the responsibilities of daily life? |
| Recovery/Mutual recovery | | | [If applicable]: How do you think this has helped you deal with (condition)? What do you think it is about the sessions that helps in that?  How was the experience of having people from so many different backgrounds here? |
| Overall wellbeing | | | Taking all things together, how happy would you say you are these days? |

*Appendix 3. Focus group protocol*

| **Introduction** | Welcoming participants; introductions  Explaining purpose and context of the focus group (no right or wrong answers, keeping confidentiality within the group)  Explaining about the research project and ethics, that information is confidential and no names will be used |
| --- | --- |
| **Main body** | General wellbeing evaluation: How would you rate your wellbeing these days?  Evaluation of the programme: What is your general evaluation of this programme?  How did this programme affect the way you feel day-to-day?  How did you experience doing music as part of this particular group?  [If applicable] Recovery: How does the making of music help us in this change ?  Transition: Of all the things we discussed, what do you think is the most important aspect to take from this experience? |
| **Close** | Thanks; contact information for further follow up; explain how data will be used |
